# Prevalence and the Associated Factors of Erectile Dysfunction Among Saudi Married Males

**DOI:** 10.7759/cureus.30998

**Published:** 2022-11-02

**Authors:** Talal M Alenezi, Shaik Shaffi Ahamed, Hussam R Almutairi, Mohammad F Aleisa, Mubashir M Alasmari, Ahmed A Bagaies, Turki H Albinhar

**Affiliations:** 1 Medicine, King Saud University, Riyadh, SAU; 2 Family & Community Medicine, King Saud University, Riyadh, SAU

**Keywords:** saudi arabia, prevalence, age, associated factors, erectile dysfunction

## Abstract

Objectives: To estimate the prevalence of erectile dysfunction (ED) and to determine the associated factors of erectile dysfunction among Saudi married male subjects.

Methods: A cross-sectional study based on data collected from 313 male subjects. The questionnaire was given to the randomly selected subjects from the appointment list in KKUH outpatient clinics using a simple random sampling method. The study questionnaire included the following sections: demographic, lifestyle, and medical history. The international index of erectile function-15 (IIEF-15) scale was used to assess ED. Statistical analyses were done using Statistical Package for Social Sciences (SPSS, IBM Corp., Armonk, NY, USA).

Results: Out of 313 participants 33 had ED (10.53%). The associated factors were age (odds ratio= 14.4), lower education level (odds ratio= 15.85), mild physical activity (odds ratio= 7.69), and medical conditions like diabetes mellitus, hypertension, and hypercholesteremia (odds ratio= 3.6).

Conclusion: The diagnosis of ED in Saudi Arabia is underestimated and services for the diagnosis and treatment should be provided for diabetes, hypertension, hypercholesteremia and elderly patients.

## Introduction

Erectile dysfunction (ED) is considered an increasing health concern that has a significant impact on the quality of life of both partners due to lower sexual satisfaction [1,2]. Erectile dysfunction is defined as the inability to keep or achieve an erection to perform a sexual interaction that satisfies both partners [3]. It is also known as a predictor of dementia, cardiovascular disease, and all-cause mortality [4,5]. The major risk factor associated with ED is age [6] and many studies show other associated factors like depression, diabetes, hypertension, use of antihypertension drugs, hypercholesteremia, atherosclerosis, and lower income and education level [7-10]. Although the high demand for clinical services and the potential impact of ED and other sexual disorders on quality of life, the epidemiological data are relatively insufficient, underreported, and undertreated in many developing countries because it is not life-threatening [11-13]. Also, some men feel ashamed of having this problem and they do not seek help [13]. Worldwide the prevalence of ED ranges from 2% in men younger than 40 years to 86% in men 80 years or older [14]. The economic burden has increased for example in the USA where the annual expenditure on ED was $330 million in 2000, compared to $185 million in 1994 [15]. The international index of erectile function (IIEF) is a tool developed to detect treatment effects in clinical trials and has excellent reliability, validity, and sensitivity in evaluating erection [16]. Finally, due to the lack of information about how common ED is among the general population of Saudi Arabia and since ED affects the quality of life of patients, through this study we aim to estimate the prevalence of erectile dysfunction among Saudi married male subjects and to determinate the associated factors of ED among these subjects.

## Materials and methods

Study sample

We obtained approval from the Institutional Review Board at King Saud University (KSU) College of Medicine (approval number: E-19-4403). After explaining the purpose of the study, verbal or written consent was taken from participants before filling out the questionnaire. Names or any identifying information were not requested from the participants and we insured that all data collected will be used only for this research. The study design is quantitative cross-sectional. The study sample consisted of patients who had an appointment in the outpatient clinics at King Khaled University Hospital (KKUH) and used a simple random sampling method. All the participants were Saudi, married, and aged 22 and above. We excluded those who are not married as we believe it will not be feasible to evaluate ED among divorced or single men due to religious and cultural barriers. Also, we excluded males aged less than 22 or those without appointments. The study was conducted from 1st January 2020 to 13th March 2020. The sample size was 313 and it was determined by using a single proportion sample size formula, N = (Zα)2 p(1-p)/d2. The sample proportion was assumed as 18.8% based on the literature with a 95% confidence level and a 5% margin of error.

Data collection tool

Our questionnaire featured social demographic, lifestyle, and medical history sections and we used a validated Arabic version of the IIEF-15 where some items were scored from 0-5 and others scored 1-5. This questionnaire assessed five functional domains, six items on erectile function (EF), two items on orgasmic function (OF), two items on sexual desire (SD), three items on intercourse satisfaction (IS), and two items on overall satisfaction (OS). The max score of EF is 30. The maximum for IS is 15, and 10 for the other domains. For the social demographic section, we had five different age groups: 22 to 34, 35 to 44, 45 to 54, 55 to 64, and above 65 years. The options for education level were as follows: no education, primary school, high school, and university graduate. As for the monthly income we measured it in Saudi riyal and divided them into the following groups: 0 to 4999, 5000 to 9999, 10000 to 14999, 15000 to 19999, and above 20000.

The lifestyle section contained two items, namely low/moderate/high physical activity, and smoker/nonsmoker/ex-smoker. Finally, For the medical history section, we were interested to know if the participant had any of the following conditions: diabetes mellitus, hypertension, and hypercholesterolemia, and if he had been diagnosed with that condition for less than a year or more.

Data collection

A pilot study was carried out on the feasibility and response of subjects. All the participants were informed about the purpose of the study and assured that their information will be confidential. Before handing out the survey, the participants were asked to confirm if they were married. The respondents were asked to give information about their social demographic status, lifestyle, and medical history.

Measurement of outcome variables

The prevalence of ED was analyzed in all 313 participants and considering the ones who had EF score of less than 14 as patients with severe ED. The effect of our study variables (age, education level, number of children, income, physical activity, smoking, diabetes mellites, hypertension, and hypercholesteremia) on sexual function was analyzed. Computing the mean score for all the domains based on age groups and comparing the erectile function domain with the other domains were also done.

Statistical analysis

Data were analyzed using the Statistical Package for Social Sciences (SPSS) version 21 (IBM Corp., Armonk, NY, USA). Descriptive statistics (frequencies, percentages, odds ratio, mean and standard deviation) were used to describe socio-demographic characteristics and clinical features. Student's T-test and analysis of variance (ANOVA) were used to perform the univariate analysis. A p-value of < 0.05 and 95% confidence intervals (CI) was used to report the statistical significance and precision of the results.

## Results

Distribution of socio-demographic characteristics and clinical features

A total of 313 respondents participated in the study. As shown in Table 1, respondents aged from 22 to > 65 with the majority of them between 35 to 44 years (32.3%). More than half have children and most have more than four children (28.8%). Of the respondents, 73.2% have a university degree. More than half i.e., 56.2%, have an Income of 10000 to 20000. Moderate physical activity was observed to be the highest (70.9%). Almost half the respondents were non-smokers (53.4). Around 26.2% have diabetes mellitus, 21.7% have hypertension, and 17.9% have hypercholesteremia, while the majority have no comorbidities (57.8%).

**Table 1:** Distribution of demographic characteristics and clinical features in 313 respondents

Variables	N	Percentage (%)
Age		
22-34	62	19.8
35-44	101	32.3
45-54	76	24.3
55-64	52	16.6
>65	22	7.0
No. of Children		
No children	25	8.5
1	33	10.5
2	54	17.3
3	52	16.6
4	59	18.8
>4	90	28.8
Education		
School Level	19	6.1
High School	65	20.8
University degree	229	73.2
Income		
<10000	110	35.1
10000-20000	176	56.2
>20000	27	8.6
Physical activity		
Mild	51	16.3
Moderate	222	70.9
Intense	40	12.8
Smoking		
Smoker	117	37.4
Not Smoker	167	53.4
Ex-Smoker	29	9.3
Comorbidities		
Diabetes mellitus	82	26.2
Hypertension	86	21.7
Hypercholesteremia	56	17.9
No comorbidities	181	57.8

The IIEF score and age groups

The mean score and the standard deviation of each IIEF-15 domain based on age groups (as shown in Table 2) with a 10-year gap between groups ranged from 22 to > 65 as follows: the mean of the EF domain ranged from 25.05 ± 4.78 to 10.45 ± 8.69, OF domain ranged from 8.37 ± 2.44 to 3.5 ± 3.86, the SD domain ranged from 8.35 ± 1.15 to 5.67 ± 1.59, the IS ranged from 10.76 ± 2.7 to 4.14 ± 3.83, and OS ranged from 9.03 ± 1.21 to 5.91 ± 1.57. The scores of all the domains decreased with age.

**Table 2:** Scoring of IIEF-15 domains based on age distributions IIEF-15: International index of erectile function, M: Mean, SD: Standard deviation

Scoring of IIEF-15 domains					
	Erectile Function	Orgasmic Function	Sexual Desire	Intercourse Satisfaction	Overall Satisfaction
Age	M ± SD				
22-34	25.05 ± 4.78	8.37 ± 2.44	8.35 ± 1.15	10.76 ± 2.7	9.03 ± 1.21
35-44	24.19 ± 4.77	8.27 ± 2.28	7.85 ± 1.26	9.95 ± 2.44	7.97 ± 1.89
45-54	21.51 ± 4.88	7.75 ± 1.92	7.17 ± 1.61	9.32 ± 2.16	7.62 ± 2.01
55-64	17.88 ± 6.07	6.35 ± 2.35	6.35 ± 1.52	7.62 ± 2.25	6.6 ± 2.12
>65	10.45 ± 8.69	3.5 ± 3.86	5.67 ± 1.59	4.14 ± 3.83	5.91 ± 1.57

Prevalence of ED and the association with socio-demographic characteristics and clinical features

Table 3 shows the association of sociodemographic and clinical features with ED. We found that the overall prevalence of ED is 10.54% of all responders (33 of 313). However, the prevalence of ED among the responders who were aged more than 55 years is 32.43% (24 of 74) and with an odds ratio (OR) of 14.4. Also, we observed that 52.6% of whom have only a school degree is suffering from ED. While 33.3% of responders who do mild physical activity and 17.4% of responders who are suffering from medical conditions have ED with OR 7.69 and 3.6, respectively. Finally, 10.25% of responders who smoke have ED. However, it is statistically insignificant with OR 1.05 (95% CI, 0.5-2.22).

**Table 3:** The prevalence of ED and its association with age, physical activity, medical conditions, smoking, and education. ED: Erectile dysfunction, OR: Odds ratio, CI: Confidence interval

	Men with ED		Men without ED		
	N	%	N	%	OR (95% CI)
Overall Prevalence	33	10.54	280	89.46	
Age					
<35	2	3.2	60	96.8	1
35-54	7	4.12	170	95.88	1.235 (0.25-6.11)
>55	24	32.43	50	67.57	14.4 (3.22-63.93)
Physical Activity					
Mild	17	33.3	34	66.7	7.69 (3.56-16.62)
Moderate to intense	16	6.1	246	93.9	1
Medical Condition					
No	10	5.5	171	94.5	1
Yes	23	17.4	109	82.6	3.6 (1.65-7.87)
Smoking					
Yes	12	10.26	105	89.74	1.05 (0.5-2.22)
No	21	9.33	175	90.66	1
Education					
School level	10	52.6	9	47.4	15.85 (5.59-44.92)
High school	8	12.3	57	87.7	2 (0.8-4.95)
University degree	15	6.6	214	93.4	1

Erectile dysfunction and other IIED domains:

Table 4 shows the comparison of ED with the sexual domains. The OF had a mean of 2.33 and a difference of -5.79 (p-value <0.001, 95% CI, 6.70-4.87). Sexual desire with a mean of 5.3 and mean difference of -2.33 (p-value <0.001, 95% CI, -3.05-1.60). Intercourse satisfaction had a mean of 3.33 and difference of -6.51 (p-value <0.001, 95% CI, -7.57-5.46). Overall satisfaction had a mean of 4.82 and a difference of -3.25 (p-value <0.001).

**Table 4:** Comparsion between erectile dysfunction and sexual domains ED: Erectile dysfunction, OF: Orgasmic function, SD: Sexual desire, IS: Intercourse satisfaction, OS: Overall satisfaction

	Mean ED	Mean non-ED	Mean difference	T-value	P-value	95% (lower-upper)
OF score	2.33	8.12	-5.79	-12.77	<0.001	-(6.70 – 4.87)
SD score	5.3	7.63	-2.33	-6.53	<0.001	-(3.05 – 1.60)
IS Score	3.33	9.85	-6.51	-12.49	<0.001	-(7.57 – 5.46)
OS score	4.82	8.06	-3.25	-8.91	<0.001	-(3.99 – 2.51)

## Discussion

Erectile dysfunction is known as difficulties in achieving an erection before sexual intercourse and maintaining it. This cross-sectional survey reported that the overall prevalence of ED in Saudi married males is 10.45%. Age was the primary variable that correlates with ED; the majority were more than 55 years old (OR 14.4) with ED. A study conducted in Iran using a self-administered questionnaire reported an ED with 18.8% prevalence that increased with age from 6% in the 20 to 39 age category to 47% in those aged 40 to 60 [8]. Another study was conducted in Turkey with a study group aged >40; it reported 33% of ED with the majority (82.9% for those aged ≥70) [10]. In a study conducted in New Zealand on men aged 40 to 70 years with the use of the IIEF, the prevalence of ED was 42% and the age-adjusted prevalence was 38% with the highest being 60% in the 60s age group [17]. In our study, the socio-demographic data analysis showed that school-level education is the most important factor which correlates with the likelihood of ED with an OR of 15.85. Followed by those aged above 55 years old with an OR of 14.4. The reason for the high percentage is the small sample size as the overall participant with school-level education were 19 and 10 of them have ED. In a study conducted in Iran, 48.8% of men with ED had only a primary school education (225 out of 461) [8]. With that said, our results suggest people with a high level of education are less likely to have ED. It may suggest that those with higher education have a better lifestyle and are physically fit. No association between cigarette smoking and ED was noticed in our study. However, some studies report the association between smoking cigarettes and ED such as an Australian study conducted in 2006 that showed that smoking cigarettes has a clear association with ED. And after adjusting for any other confounding factors, this association was further reinforced as the number of cigarettes smoked increased. Compared with non‐smokers, the adjusted OR for ED was 1.24 (95% CI, 1.01-1.52, p=  0.04) for those smoking <20 cigarettes per day, and 1.39 (95% CI, 1.05-1.83, p =  0.02) for those smoking >20 cigarettes per day [18]. As for chronic health conditions such as diabetes mellitus, and hypertension and their association with ED, a systematic review and meta-analysis of 145 studies conducted in 2016 showed that diabetic men had a prevalence of approximately 3.5 times more than controls [19]. Another study conducted in the USA showed that ED has a higher prevalence in patients with hypertension than those reported in an age-matched general population and that ED is more severe in patients with hypertension than in the general population [20].

Our study provides an estimation of ED prevalence in Saudi Arabia among the married male population and addresses the possible factors that might be associated with it. However, our study does not provide information about non-married males or previously married male subjects as we limited our study to only those who are currently married. Also, our study lacks details about the psychological factors that could be associated with ED as well as the desire of getting treated for ED.

## Conclusions

We conclude the following from our study: the prevalence of ED among Saudi married men is 10.53%; the factors associated with ED are age, medical condition, physical activity, and a lower education level. However, in our study, smoking was not statistically significant. We recommend services for the diagnosis and treatment of ED, especially for those who are diabetic, hypertensive, or elderly in primary care clinics, as the diagnosis of ED in Saudi Arabia is underestimated. 
